# Telemetric monitoring of intracystic pressure with sensor reservoir for recurrent sellar arachnoid cysts

**DOI:** 10.1016/j.bas.2022.100874

**Published:** 2022-02-22

**Authors:** Gianpaolo Jannelli, Emmanuel Jouanneau, Romain Manet

**Affiliations:** Skull Base and Pituitary Neurosurgical Department, Hôpital Neurologique Pierre Wertheimer, Claude Bernard University, Lyon, France; Skull Base and Pituitary Neurosurgical Department, Hôpital Neurologique Pierre Wertheimer, Claude Bernard University, Lyon, France; Lyon 1 University, Lyon, France; INSERM U1052, CNRS, UMR5286, Cancer Research Center of Lyon, Lyon, France; Skull Base and Pituitary Neurosurgical Department, Hôpital Neurologique Pierre Wertheimer, Claude Bernard University, Lyon, France

**Keywords:** SAC, Sellar arachnoid cyst

## Abbreviation

SAC: Sellar arachnoid cysts

Dear Editor,

Sellar arachnoid cysts (SAC) are rare (3% of all intracranial arachnoid cysts) but represent serious conditions due to their possible visual consequences resulting from opto-chiasmatic compression. Their surgical management remains controversial. A recent article from d'Artigues et al. reported a series of 17 patients (the largest series published to date) diagnosed with SAC and discussed their management that can be challenging (d'Artigues et al., 2021). They performed, in all cases, a fully endoscopic transsphenoidal approach, and reconstructed the sella using abdominal fat. As McLaughlin et al. stated in their previous series, the fat seems to play a promising role in preventing two main complications of this surgery (McLaughlin et al., 2012). On one hand, it represents a “barrier” able to obstruct the communication between the subarachnoid space and the cyst. The result is a lower risk of immediate CSF leak compared to previous series. On the other hand, the fat could induce scar formation to the diaphragma and parasellar arachnoid, preventing the sella from refilling with CSF. However, the long terme outcome remains unclear.

Given the findings from our own clinical practice (series of approximately 2500 pituitary surgeries of the senior author, EJ), we encourage the use of endoscopic endonasal approach as the first line treatment for this rare entity. However, the management of recurrences and in particular the decision-making process in case of failed endoscopic or transcranial fenestration is still debated. We personally faced two cases of recurrence that we managed with the insertion of an intracystic catheter, introduced by a neuroendosccopic transventricular approach and connected to a subcutaneous reservoir system. This strategy seems to be promising in the treatment of other cystic sellar lesions, such as recurrent craniopharyngiomas not eligible for gross total resection (Frio et al., 2019). The more informative of these cases is the last one.

**Fig. 1:**
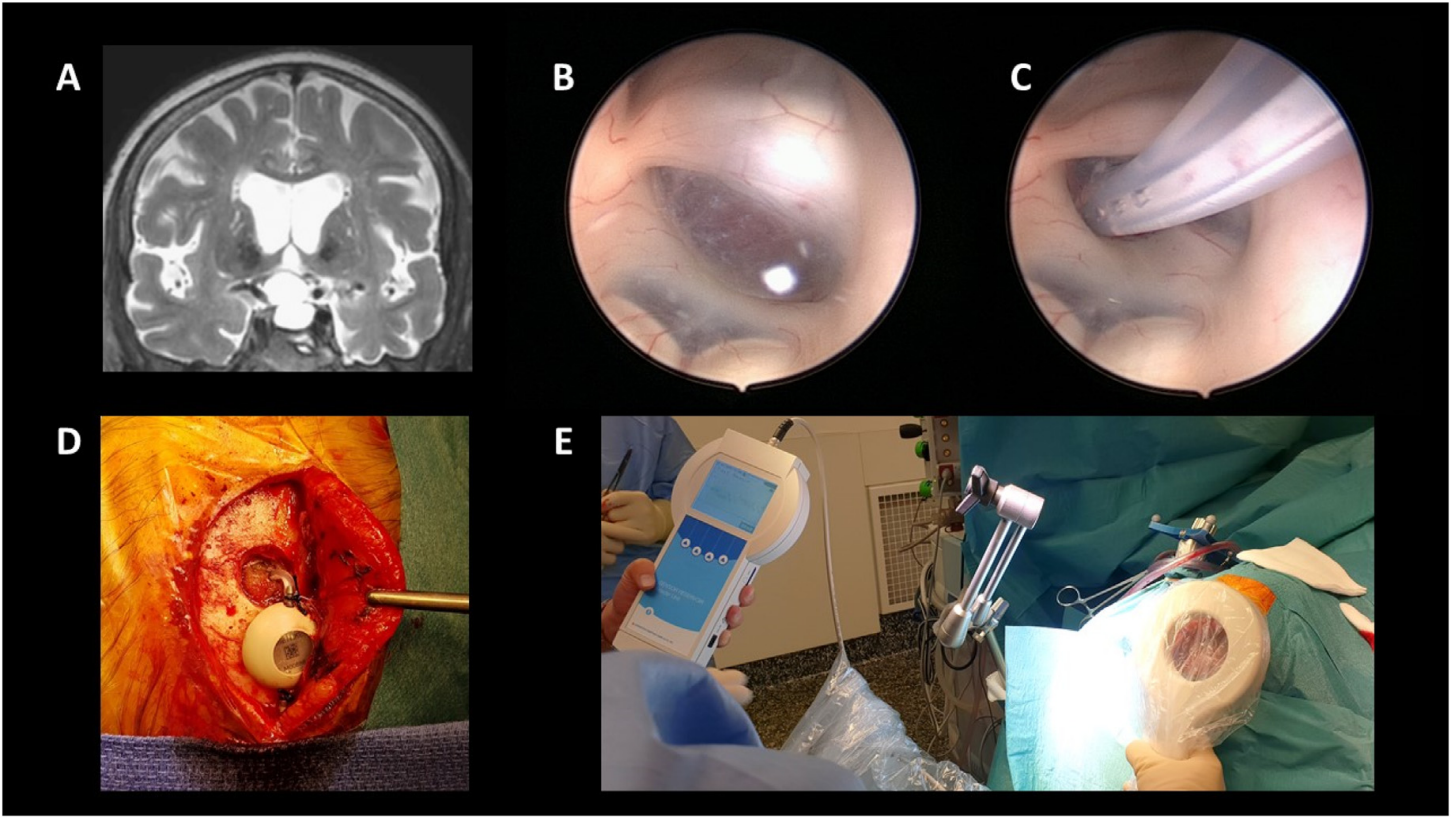
A: Pre-operative MRI showing chiasmatic stretching related to suprasellar extension of the arachnoid cyst. B: Per-operative view of the cyst before fenestration. C: Per-operative view after cystic fenestration and insertion of the catheter. D: Per-operative view showing the Sensor Reservoir. E: Per-operative checking of the effectiveness of telemetric recording of intracystic pressure.

A 77 years-old patient complained of visual field defect due to a SAC compressing optic chiasma with severe chronic visual impairment. We initially decided to treat the cyst by an endoscopic endonasal approach, without reconstructing the sella with the fat (no CSF leak). Two months later, the patient presented a CSF rhinorrhea, requiring a second intervention to repair the leakage with fat. At one-year follow-up, the patient worsening once again visually. The MRI showed a recurrence of the cyst. At that time, we decided to perform a large fenestration of the cyst in the suprasellar subarachnoid space by a transcranial approach. Unfortunately, one year later, the patient complained of a new visual field worsening upon the second occurrence of cystic regrowth (Fig. 1 a).

Due to failure of previous standard strategies, we decided to place a catheter in the cyst through an endoscopic transventricular approach (Fig. 1 b, c). Instead of a classic Omaya reservoir, we connected the catheter to a Sensor Reservoir ® (Miethke, Aesculap, Germany, Fig. 1 d). This device, designed for management of complex CSF disorders, allows a telemetric measurement of intracranial pressure (Antes et al., 2018). Two considerations supported our decision. First, as a classic Omaya reservoir, it allows CSF withdrawal through a percutaneous puncture. Second, the possibility to directly measure non-invasively the pressure within the cyst would allow to anticipate clinical consequence of its rising (i.e. visual worsening), as morbidity is related to the pressure of the cyst as much as its volume. As soon as pressure is confirmed to be increased, a CSF withdrawal through the reservoir may anticipating visual worsening.

At the end of the procedure, we checked the good functioning of the device with per-operative telemetric measurement of the pressure (pulsatile signal - mean pressure: 5 ​mmHg in semi-sitting position) (Fig. 1 e). The procedure was uneventful and the patient was discharged at day 6 with a stabilized vision. For the moment, the device does not allow continuous measurement. We therefore decided to carry out measurements during routine follow-ups and in any event of a subjective sensation of visual change reported by the patient. Continuous measurement of the pressure might improve decision making and especially our understanding of this condition.

One year later, he presented with a new visual worsening. A control of the pressure revealed an increased value (11 ​mmHg), secondly confirmed by a new increased of cystic volume on MRI, and CSF withdrawal was achieved by direct percutaneous puncture within the reservoir. This maneuver allowed a normalization of the pressure to its initial value (5 ​mmHg) and a stabilization of the vision, maintained at two-years follow up.

To the best of our knowledge, this is the first case of sellar arachnoid cyst treated by this technique. Due to its high price and the technicality of its use, its routine placement couldn't be advocated but we would like to suggest this strategy for selected cases of failure of endoscopic and/or open approaches of recurrent SAC. Normal and pathological values of intracystic pressure remain to be determined by further studies.

### Patient consent

The patient consented to publication.

### Ethical approval

No ethical approval was required for this type of study.

## Declaration of competing interest

The authors declare that they have no known competing financial interests or personal relationships that could have appeared to influence the work reported in this paper.
